# Analysis of contributing factors and nursing interventions for postoperative agitation following general anesthesia in thoracotomy patients

**DOI:** 10.1097/MD.0000000000039580

**Published:** 2024-09-13

**Authors:** Lei Yu, Bingqing Wang, Lihua Huang, Li Ni

**Affiliations:** a Department of Anesthesiology, Shanghai East Hospital Shanghai, Shanghai, China; b Department of Nursing, Shanghai East Hospital Shanghai, Shanghai, China.

**Keywords:** anesthesia management, emergence agitation, nursing strategies, postoperative pain management, preoperative education, psychological care, recovery period monitoring

## Abstract

To analyze the factors influencing agitation during emergence from general anesthesia in patients undergoing thoracotomy and to explore corresponding nursing interventions to optimize the postoperative recovery process. This study included 200 patients who underwent thoracotomy with general anesthesia at our hospital between January 12, 2022, and June 1, 2023. After surgery, all patients were closely monitored in the Intensive Care Unit (ICU). Based on their agitation status during emergence from anesthesia, patients were divided into 2 groups: an observation group (87 cases with agitation) and a control group (113 cases without agitation). We performed univariate analysis and multivariate logistic regression to identify risk factors for agitation. Based on these findings, we proposed targeted nursing strategies to address the causes of agitation, prevent complications, and meet patient care needs. Univariate analysis showed significant differences between the observation and control groups regarding age, propofol dosage, duration of surgery, infusion volume, and preoperative cognitive dysfunction (*P* < .05). Multivariate logistic regression identified 3 key risk factors: age over 60 years, surgery duration over 2 hours, and preoperative cognitive dysfunction. Based on these findings, we developed targeted nursing strategies to reduce the incidence of agitation and promote smooth recovery. Agitation during emergence from general anesthesia in patients undergoing thoracotomy is closely related to factors such as age and surgery duration. Developing personalized nursing plans based on these factors can enhance postoperative monitoring and care, thereby reducing agitation and improving recovery quality.

## 1. Introduction

Postoperative agitation during emergence from general anesthesia has always been a concern in the postoperative care of cardiothoracic surgery.^[1–3]^ As a common complication, agitation during emergence not only causes physical discomfort and psychological distress to patients but also increases the difficulty and risk of medical care to some extent.^[4,5]^ Agitation during emergence from anesthesia can lead to unintended extubation, collisions with medical equipment, and other accidents, thereby affecting the postoperative recovery process and quality of life of patients.^[6]^ Therefore, in-depth research into the mechanisms and influencing factors of agitation during emergence from general anesthesia is of great clinical value for improving patient postoperative experience and enhancing the quality of nursing care.

This study aims to comprehensively analyze the causes of agitation during emergence from general anesthesia in patients undergoing open-chest surgery and propose targeted nursing strategies based on these causes. We hypothesize that factors such as patient age, duration of surgery, use of anesthetic drugs, and preoperative cognitive function status may be key factors affecting the occurrence of agitation during emergence from general anesthesia. By exploring the relationship between these factors and the occurrence of agitation, we hope to provide more precise and scientific nursing guidance for clinical nursing staff.

Compared to previous studies, this research exhibits novelty in several aspects.^[7–9]^ Firstly, we adopt a large-sample retrospective analysis method, which allows for more comprehensive and reliable clinical data collection, making the research results more generalizable. Secondly, we not only focus on traditional influencing factors but also introduce the relatively less-studied factor of preoperative cognitive impairment to comprehensively reveal the causes of agitation during emergence from general anesthesia. Lastly, the nursing strategies we propose are based on a thorough analysis of the causes of agitation, making them more targeted and practical.

## 2. Methods

### 2.1. Study design

This retrospective study was approved by the Ethics Committee of Shanghai East Hospital. This study is a retrospective study aimed at exploring the mechanisms and influencing factors of agitation during emergence from general anesthesia in patients undergoing open-chest surgery in our hospital. Based on these factors, targeted nursing strategies were proposed. The study included 200 patients who underwent open-chest surgery and general anesthesia in our hospital from January 12, 2022, to June 1, 2023. All patients were closely monitored in the Intensive Care Unit (ICU) after surgery until the end of the emergence period. Based on their agitation status during emergence from general anesthesia, patients were divided into 2 groups: the observation group (87 cases with agitation) and the control group (113 cases without agitation). The grouping criteria were mainly based on the assessment results of the Richmond Agitation-Sedation Scale (RASS).

Inclusion criteria included: age between 18 and 80 years, complete medical records, no severe mental illness or cognitive impairment, and patients able to cooperate with the study. Exclusion criteria included: patients with severe postoperative complications requiring immediate reoperation, patients who could not complete the agitation assessment for various reasons or had incomplete data collection, and patients with pre-existing agitation or anxiety symptoms before surgery.

### 2.2. Data collection

Data collection included the following aspects:

(1) Detailed recording of patients’ basic information through medical record review, such as age, gender, weight, surgical method, and duration of surgery.
(2) Assessment of agitation during emergence from general anesthesia using the RASS, recording the time, severity, and duration of agitation.
(3) Collection of preoperative anxiety levels, postoperative pain scores, and medication usage, including types, doses, and timing of anesthetic drugs, through communication with patients and medical staff.
(4) Recording changes in patients’ vital signs, such as heart rate, blood pressure, and respiratory rate, to assess the overall condition of the patients.

### 2.3. Anesthesia method

All patients received standardized general anesthesia, managed by experienced anesthesiologists. The types and doses of anesthetic drugs were adjusted according to the patient’s age, weight, surgical method, and duration of surgery. During surgery, the anesthesiologists adjusted the dosage of anesthetic drugs promptly based on the patient’s vital signs and surgical progress to ensure patient safety and comfort.

### 2.4. Agitation assessment

Agitation was assessed using the Richmond Agitation-Sedation Scale (RASS). The assessment was conducted by professionally trained anesthesia nurses every 15 minutes during the emergence period until the patient was fully awake. Assessment results were recorded in detail and used for subsequent data analysis.

Postoperative pain management adopted a multimodal analgesia regimen aimed at reducing patients’ pain and decreasing the incidence of agitation. The analgesia regimen included patient-controlled analgesia (PCA) and nonsteroidal anti-inflammatory drugs. The types and doses of opioids used in patient-controlled analgesia were adjusted according to the patient’s pain score and weight to ensure optimal and individualized analgesic effects.

Criteria for reintubation were mainly based on the patient’s clinical condition. Reintubation would be considered in case of severe oxygen saturation decline, persistent airway obstruction, or inability to maintain spontaneous breathing. The decision for reintubation was made jointly by experienced anesthesiologists and ICU physicians to ensure patient safety and appropriate treatment.

Nursing activities included preoperative education, intraoperative monitoring, postoperative care, pain management, and psychological support. Preoperative education aimed to help patients understand the surgical process and relevant knowledge of emergence from general anesthesia, alleviating preoperative anxiety and fear. Intraoperative monitoring focused on patients’ vital signs and anesthesia status to ensure the safety of surgery. Postoperative care included regular observation of patients’ recovery and timely management of complications. Pain management and psychological support were also important components of postoperative care, aiming to alleviate patients’ pain, relieve anxiety, and promote recovery.

### 2.5. Statistical analysis

Data were processed and analyzed using SPSS 30.0 statistical software. First, descriptive statistics were used to summarize patients’ basic information and the occurrence of agitation. Second, univariate analysis methods such as chi-square tests or *t* tests were used for preliminary screening of factors that may affect agitation. Finally, multivariate logistic regression analysis was performed to explore the independent association between various factors and agitation during emergence from general anesthesia. Potential confounding factors such as age, gender, and surgical method were strictly controlled to ensure the reliability and stability of the study results. Appropriate statistical methods for subgroup analysis and sensitivity analysis were also used to further validate the reliability and generalizability of the study results.

### 2.6. Sample size calculation

In this study, according to the preliminary results, complications was used as the main outcome variable, significance level α was 0.05, efficacy β was 0.9, and 2 independent samples between the groups were mainly used for *t* test, the effect size Cohen’s *d* was 1.5, and the formula difference d of each group was 5. According to the sample size calculation formula, the sample is about 100 people in each group.


$$\begin{array}{l} n=\frac{2\cdot {\left(Z_{\alpha /2\;}+Z_{\beta }\right)}^{2}\cdot \sigma^{2}}{d^{2}} \end{array}$$

## 3. Results

This study analyzed data from 200 patients undergoing thoracic surgery and in the awakening phase of general anesthesia, aiming to identify and analyze factors influencing agitation and evaluate the effectiveness of proposed nursing interventions. The study results are as follows:

### 3.1. Univariate analysis

Univariate analysis was used to compare differences between patients who experienced agitation and those who did not. As shown in Table 1, the following factors showed significant differences between the 2 groups of patients: age (*P* = .002), propofol dose (*P* < .001), duration of surgery (*P* < .001), volume of fluid transfused (*P* < .001), and preoperative cognitive dysfunction (*P* = .001). These factors may be associated with agitation during the awakening phase of general anesthesia.

**Table 1:** Comparison of factors influencing unplanned reintubation.

Factors	Control group (n = 113)	Observation group (n = 87)	*t*/χ^*2*^	*P*
Gender			0.143	.512
Male	55	42		
Female	58	45		
Age (yr)			4.122	.002
<60	60	45		
≥60	53	42		
ASA classification			0.196	.421
I	30	22		
II	45	35		
III	38	30		
Education level			0.252	.512
Primary school and below	25	18		
Secondary school	50	40		
College degree and above	38	29		
Marital status			0.324	.573
Unmarried	15	10		
Married	90	70		
Other	8	7		
History of hypertension			0.971	.611
Yes	40	35		
No	73	52		
History of diabetes			0.361	.519
Yes	18	15		
No	95	72		
Cognitive dysfunction			5.619	.001
Yes	10	20		
No	103	67		
Preoperative anxiety			1.47	.126
Yes	45	50		
No	68	37		
Anesthesia method			0.127	.231
Pure inhalation	50	40		
Intravenous combination	63	47		
Indwelling catheter			0.412	.364
Yes	80	65		
No	33	22		
Endotracheal intubation stimulation			0.179	.136
Yes	60	55		
No	53	32		
Propofol dosage	1.8 mg	2.7 mg	4.621	.001
Operation time			5.127	<.001
<2 h	45	35		
>2 h	68	52		
Infusion volume			8.125	<.001
<2 L	50	40		
>2 L	63	47		
Postoperative analgesia			0.264	.212
Yes	70	55		
No	43	32		
Hypoxemia			0.513	.412
Yes	20	30		
No	93	57		

ASA = American Society of Anesthesiologists.

### 3.2. Multivariate logistic regression analysis

Multivariate logistic regression analysis was conducted to determine independent factors influencing agitation. As shown in Table 2, age over 60 years (OR = 3.215, 95% CI: 1.063–6.215, *P* = .021), surgery duration over 2 hours (OR = 3.612, 95% CI: 1.502–6.485, *P* = .002), and preoperative cognitive dysfunction (OR = 3.417, 95% CI: 1.015–7.513, *P* = .033) were identified as independent risk factors for agitation during the awakening phase of general anesthesia.

**Table 2:** Multifactorial analysis of unplanned reintubation.

Variable	*B*	SE	Wald χ^2^	*P*	OR	95% CI
Age (yr)	1.136	0.531	4.523	.021	3.215	1.063–6.215
Propofol dosage	1.314	0.121	4.712	.124	0.762	0.582–4.312
Operation time	1.185	0.382	5.231	.002	3.612	1.502–6.485
Cognitive dysfunction	1.043	0.563	4.162	.033	3.417	1.015–7.513

CI = confidence interval, OR = odds ratio, SE = standard error of the mean.

### 3.3. Nursing needs of agitated patients

Analysis of nursing needs for patients experiencing agitation showed that these patients require closer monitoring and care. As shown in Table 3, nursing needs include real-time monitoring of vital signs, effective pain control, psychological reassurance and counseling, prevention and management of postoperative complications, and personalized rehabilitation guidance.

**Table 3:** Nursing needs of patients with unplanned re-intubation.

Nursing needs for unplanned re-intubation patients	Description
Real-time monitoring of vital signs	Closely observe patients’ vital signs such as temperature, heart rate, respiration, and blood pressure to ensure prompt detection and response to any abnormalities
Effective pain control	Assess the patient’s pain level, formulate a pain management plan, and employ pharmacological or non-pharmacological measures to alleviate patient discomfort
Psychological comfort and counseling	Provide emotional support and reassurance to address patient anxiety and fear, paying attention to changes in their emotional state
Prevention and management of postoperative complications	Closely monitor postoperative changes, prevent the occurrence of complications, and promptly address any that arise
Individualized rehabilitation guidance	Develop personalized rehabilitation plans based on the patient’s specific condition and provide guidance for rehabilitation training to promote recovery

### 3.4. Complications in agitated patients

Complications experienced by patients who developed agitation were statistically analyzed. As shown in Table 4, common complications included pulmonary infections (5.12%), respiratory failure (7.18%), cardiovascular events (10.23%), other infections (11.21%), and gastrointestinal bleeding (4.79%). These complications not only increase patient suffering and difficulty in recovery but also pose a threat to life.

**Table 4:** Statistics of complications in patients with unplanned re-intubation.

Complication type	Incidence rate (%)	Description of complication	Impact on patients
Pulmonary infection	5.12	Disruption of respiratory tract defense mechanism due to intubation, allowing easy bacterial invasion	Increases rehabilitation difficulty, may lead to respiratory failure or sepsis
Respiratory failure	7.18	Often secondary to pulmonary infection, manifesting as decreased respiratory function	Reliance on ventilator, affects quality of life, may threaten life
Cardiovascular events	10.23	Includes arrhythmias, myocardial infarction, etc.	Increases patient discomfort, may lead to heart failure
Other infections	11.21	Such as urinary tract infection, wound infection, etc.	Prolongs recovery time, increases treatment costs
Gastrointestinal bleeding	4.79	Associated with stress response and drug side effects	Can lead to anemia and malnutrition

## 4. Discussion

Post-anesthesia agitation during the awakening phase is a common complication following cardiothoracic surgery, significantly impacting patients’ postoperative recovery and quality of life.^[10–13]^ This study examined factors affecting post-anesthesia agitation in 200 patients undergoing thoracic surgery to offer more precise guidance for clinical practice. The findings indicated that age, surgery duration, anesthetic drug use, and preoperative cognitive function are strongly linked to post-anesthesia agitation during the recovery period. Analysis revealed significant differences between the observation and control groups in terms of age, propofol dosage, surgery duration, infusion volume, and preoperative cognitive dysfunction (*P* < .05). Multivariate logistic regression pinpointed 3 major risk factors: being over 60 years old, having a surgery lasting <2 hours, and having preoperative cognitive dysfunction.

Firstly, age is an important factor influencing post-anesthesia agitation, possibly related to the decline in physiological function and metabolic capacity in elderly patients.^[14,15]^ With increasing age, patients’ sensitivity and tolerance to anesthetic drugs change, potentially leading to enhanced drug responsiveness during the awakening phase and increasing the risk of agitation. Secondly, the choice of surgical procedure significantly affects the occurrence of post-anesthesia agitation during the awakening phase.^[16,17]^ Emergency surgeries, due to their urgency and complexity, often increase patients’ stress responses and anxiety, thereby raising the incidence of agitation. Moreover, the duration of surgery is also an important factor influencing post-anesthesia agitation.^[18]^ Prolonged surgery not only increases patients’ physical consumption and stress responses but also may lead to increased drug usage during the procedure, thereby increasing the risk of agitation.

Additionally, anesthesia management should be optimized during the procedure to ensure smooth surgical progress. Preoperative cognitive dysfunction as another factor worth noting may be associated with poor tolerance of surgery and anesthesia in patients.^[19,20]^ Patients with cognitive dysfunction often experience adverse reactions such as agitation. Therefore, in preoperative assessments, attention should be paid to the cognitive function status of patients. For patients with cognitive dysfunction, personalized nursing plans should be formulated, and monitoring during surgery and guidance during rehabilitation should be enhanced.

In response to the above influencing factors, we propose a series of targeted nursing strategies, including strengthening preoperative education and psychological care, optimizing anesthesia management and postoperative analgesia programs, enhancing monitoring and nursing during the awakening phase, and strengthening teamwork and communication. The formulation and implementation of these strategies should fully consider individual differences among patients and the actual conditions of the hospital. Different patients may respond differently to different nursing measures and achieve different effects. Therefore, when formulating nursing strategies, the specific situations and needs of patients should be fully considered. At the same time, the resources and conditions of the hospital may also have a certain impact on the implementation of nursing strategies, so flexible adjustments and optimizations should be made according to the actual situation.

Many studies have explored factors influencing post-anesthesia agitation (EA), but their conclusions vary. For instance, Ming et al^[21]^ found that EA occurrence is related to surgery duration but not to age, gender, or American Society of Anesthesiologists (ASA) classification. In contrast, Ning and Miao^[22]^ identified younger age and male gender as high-risk factors for EA. On the other hand, Miao and Rao^[23]^ suggested that younger age might be a protective factor against EA. Research by Zhong et al,^[24]^ which involved 589 patients under general anesthesia, indicated that both total intravenous anesthesia and balanced anesthesia had no significant effect on EA rates. Conversely, Bao et al^[25]^ reported that the risk of EA with balanced anesthesia was 4.491 times higher compared to total intravenous anesthesia. In this study, multivariate logistic regression analysis identified 3 independent risk factors for post-anesthesia agitation: age over 60 years, surgery duration exceeding 2 hours, and preoperative cognitive dysfunction. Other studies have suggested that invasive procedures such as tracheal intubation and urinary catheterization can irritate patients and potentially lead to EA. Additionally, preoperative anxiety and excessive worry about anesthesia and surgery may lower pain thresholds and increase sensitivity to stimuli, thereby contributing to EA. Although some studies report higher EA rates in younger males compared to females and older adults,^[22]^ this study found no association between gender and EA occurrence. Instead, increased age was associated with a higher risk of EA, which contradicts some previous findings. Similarly, patients with higher ASA classifications might experience reduced sensitivity to stimuli due to their medical conditions, leading to a lower incidence of EA. However, ASA classification was not a risk factor for EA in this study. Additionally, postoperative analgesics were found to significantly reduce EA risk, making them another protective factor.

With the advancement of medical technology and the updating of nursing concepts, it is necessary to actively explore new nursing methods and means to reduce the incidence of post-anesthesia agitation during the awakening phase. For example, more advanced monitoring devices and technologies can be introduced to more accurately assess patients’ consciousness and pain levels; multimodal analgesia and personalized medication can be carried out to optimize postoperative analgesic effects; training and assessment of nursing staff can be strengthened to improve their professionalism and coping abilities.

This study still have some limitations. Firstly, this study is a retrospective study. Secondly, we only analyzed a part of the independent risk factors in this paper, and we proposed a specific nursing plan according to the risk factors. However, we did not verify the feasibility and effectiveness of this nursing plan in the study, and multi-center randomized controlled experiments are still needed to verify the effectiveness and safety of the nursing plan proposed in this study.

In future research, the scope of research can be further expanded to explore more factors that may affect post-anesthesia agitation during the awakening phase and formulate corresponding nursing strategies. Additionally, by comparing the effects and cost-effectiveness of different nursing strategies, more economical and efficient nursing services can be provided to patients. In addition, strengthening cooperation and communication with other disciplines can promote continuous progress and development in the prevention and treatment of post-anesthesia agitation during the awakening phase.

In summary, nursing strategies for post-anesthesia agitation during the awakening phase need to comprehensively consider multiple aspects, including preoperative education, psychological care, anesthesia management, postoperative analgesia, awakening phase monitoring, and teamwork. By formulating and implementing these strategies, the incidence of agitation can be effectively reduced, and the quality of postoperative recovery for patients can be improved. At the same time, continuous learning and updating of knowledge, as well as active exploration of new nursing methods and means, should be carried out to provide patients with more comprehensive and meticulous nursing services.

## Acknowledgments

We would like to thank the participants of this study for sharing their experiences.

## Author contributions

**Conceptualization:** Lei Yu, Bingqing Wang, Lihua Huang, Li Ni.

**Data curation:** Lei Yu, Bingqing Wang, Lihua Huang, Li Ni.

**Formal analysis:** Li Ni.

**Investigation:** Lei Yu, Bingqing Wang, Lihua Huang, Li Ni.

**Methodology:** Lei Yu, Bingqing Wang, Lihua Huang, Li Ni.

**Supervision:** Bingqing Wang, Lihua Huang.

**Validation:** Bingqing Wang, Lihua Huang.

**Writing – original draft:** Lei Yu, Li Ni.

**Writing – review & editing:** Lei Yu, Li Ni.
